# Web-based Dashboard on ECMO Utilization in Germany: An Interactive Visualization, Analyses, and Prediction Based on Real-life Data

**DOI:** 10.1007/s10916-024-02068-w

**Published:** 2024-05-10

**Authors:** Benjamin Friedrichson, Markus Ketomaeki, Thomas Jasny, Oliver Old, Lea Grebe, Elina Nürenberg-Goloub, Elisabeth H. Adam, Kai Zacharowski, Jan Andreas Kloka

**Affiliations:** Department of Anaesthesiology, Intensive Care Medicine and Pain Therapy, Goethe University Frankfurt, University Hospital, Theodor-Stern Kai 7, 60590 Frankfurt, Germany

**Keywords:** ECMO, Medical dashboard, Healthcare policy, Critical care, Pandemic preparedness

## Abstract

In Germany, a comprehensive reimbursement policy for extracorporeal membrane oxygenation (ECMO) results in the highest per capita use worldwide, although benefits remain controversial. Public ECMO data is unstructured and poorly accessible to healthcare professionals, researchers, and policymakers. In addition, there are no uniform policies for ECMO allocation which confronts medical personnel with ethical considerations during health crises such as respiratory virus outbreaks.Retrospective information on adult and pediatric ECMO support performed in German hospitals was extracted from publicly available reimbursement data and hospital quality reports and processed to create the web-based ECMO Dashboard built on Open-Source software. Patient-level and hospital-level data were merged resulting in a solid base for ECMO use analysis and ECMO demand forecasting with high spatial granularity at the level of 413 county and city districts in Germany.The ECMO Dashboard (https://www.ecmo-dash.de/), an innovative visual platform, presents the retrospective utilization patterns of ECMO support in Germany. It features interactive maps, comprehensive charts, and tables, providing insights at the hospital, district, and national levels. This tool also highlights the high prevalence of ECMO support in Germany and emphasizes districts with ECMO surplus – where patients from other regions are treated, or deficit – origins from which ECMO patients are transferred to other regions. The dashboard will evolve iteratively to provide stakeholders with vital information for informed and transparent resource allocation and decision-making.Accessible public routine data could support evidence-informed, forward-looking resource management policies, which are urgently needed to increase the quality and prepare the critical care infrastructure for future pandemics.

## Introduction

Extracorporeal life support (ECLS) techniques are an integral part of modern intensive care medicine. Of these, veno-venous (V-V) and veno-arterial (V-A) extracorporeal membrane oxygenation (ECMO) are therapeutic options for patients with cardiac and/or respiratory failure who do not respond to conventional treatment. Due to the small number of patients worldwide and the difficulties of randomized trials in this critically ill population, the quality of evidence and specific guidelines for patient selection and treatment are limited [1, 2]. Despite the initial success of ECMO in neonatal respiratory failure, its benefit in various clinical scenarios in other populations has not been clearly demonstrated [3–9]. In addition, ECMO requires substantial financial and human resources and carries a significant risk of complications [10–12]. Both the guideline of the German Society for Thoracic, Cardiac and Vascular Surgery and a position paper of the International Extracorporeal Life Support Organization (ELSO) recommend thorough consideration of the use of ECMO by an experienced multidisciplinary team [13, 14]. High-volume multidisciplinary centers with sufficient experienced staff, regular training, and appropriate funds potentially provide the optimal setting for a safe, high-quality ECMO program. Accordingly, ECMO support in high-volume compared to low-volume centers is associated with better patient outcomes in Germany [15] and worldwide [16]. During past respiratory virus outbreaks, including severe acute respiratory syndrome (SARS), H1N1 influenza, Middle East respiratory syndrome (MERS), and the recent coronavirus disease (COVID-19) pandemic, ECMO has been used at increasing rates and with advanced technical maturity but without medical consensus [17–19]. It is particularly in such crisis situations that, in addition to the challenging work, the assignment of resources and ethical considerations weigh heavily on the shoulders of the medical staff [20, 21].

National clinical quality registries offer significant benefits to critical care medicine by facilitating the systematic collection, analysis, and interpretation of clinical and epidemiological data, promoting the identification of best practice, highlighting areas for improvement, and providing information for policy and guideline development [22]. The international ELSO registry (https://www.elso.org/registry.aspx) is an important pillar for improving the safety and efficiency of ECMO in clinical practice. It collects comprehensive medical data and outcomes of ECMO patients in participating ELSO centers worldwide and provides data for research, clinical guidelines, and benchmarking. Since the Covid-19 pandemic, the ELSO added an online availability map where individual centers could report if they had available ECMO capacities, but mainly the last updates are older than 2 years. Since April 2020, the German Interdisciplinary Association for Intensive Care and Emergency Medicine (DIVI) registry (https://www.intensivregister.de/) has been recording the occupancy of intensive care and invasive ventilation capacities in around 1,300 hospitals in Germany on a daily basis, including data on COVID-19 patient numbers. The data is displayed in interactive dashboards and has been a source of information for the public during the pandemic. However, ECMO capacity is only captured as an estimate by the reporting person and provided in traffic light format from “available” to “fully utilized”. During the COVID-19 pandemic in the United States, efforts likewise focused on the equitable distribution of ventilators [23, 24], but the key challenge transpired to be the allocation of ECMO resources, which raised ethical considerations and highlighted the lack of a standardized ECMO distribution system [25].

Although the data of ECMO usage in Germany is available from different sources, it is unstructured and not easy to interpret. We developed the ECMO Dashboard (https://www.ecmo-dash.de/) and integrated the unstructured data provided by the German Federal Statistical Office (DESTATIS) and the quality reports of the hospitals in Germany. The dashboard features easy-to-use, interactive visualization and analysis of supply and demand for adult and pediatric V-A and V-V ECMO in city and county districts, as well as the ECMO volumes in individual hospitals in Germany since 2006. Further, we included a predictive tool that enables proactive allocation of patients to ECMO centers and efficient resource management across regions and centers. The dashboard is updated regularly to make the most up-to-date available healthcare data accessible and understandable for healthcare workers, political decision-makers, researchers, and the interested public. Thereby it should provide incentives to improve the public health system through data-driven policy guidelines, medical quality assurance, and informed participation of the public.

## Materials and Methods

### Origins of Data

The information on the number of ECMO support provided in this study is based on the 2006–2021 data from the structured quality reports of the hospitals (Qualitätsberichte der Krankenhäuser). This data is collected by the German Federal Joint Committee (Gemeinsamer Bundesausschuss, G-BA). All registered hospitals in Germany are legally obliged submit a report on their work and structures on an annual basis. In 2021, there were approximately 1,914 hospitals that submitted a quality report to the G-BA. Quality reports provide hospital-specific information on, such as the range of diagnoses and treatments, the number of treatments and procedures performed, and staffing levels. No patient-specific information is provided in these reports. The data analysis as conducted in this study complies with the data use policy of G-BA. The data on the origins of patients receiving ECMO support is provided by the Institute for the Hospital Remuneration System (Institut für das Entgeltsystem im Krankenhaus, InEK) and stored and made available for remote processing by DESTATIS for the years 2005–2021. The data was queried using SAS (SAS Institute Inc., Cary, NC, USA) from the DESTATIS database. Due to data protection regulations set by DESTATIS, releasing hospital-level case data from its records is restricted. As such, ECMO instances at the hospital level were analyzed using the G-BA hospital quality reports. As the data are publicly available, the need for ethical approval was waived by the Ethics Committee of the University Hospital Frankfurt (Ref: 2022 − 766).

### Data Extraction and Development of the ECMO Dashboard

The hospital quality reports are provided in .xml file format in separate files for each available hospital. The data on relevant ECMO support were identified using the German procedure classification (Operationen- und Prozedurenschlüssel, OPS, Table 1) published by the German Federal Institute for Drugs and Medical Devices (Bundesinstitut für Arzneimittel und Medizinprodukte, BfArM) and extracted using a custom-made xml parser implemented in Python.

**Table 1:** OPS codes used for identifying relevant treatments

OPS code	Description
8-852-0	Veno-venous extracorporeal membrane oxygenation (ECMO) without cardiac support
8-852-3	Use of minimalized heart-lung device

For cases where a hospital conducted a specific treatment (including ECMO support) 1–3 times during a reporting period, the individual quality reports indicated privacy-related redaction of numerical data. In this case, a mean value of 2 was assumed for these redacted cases.

The data on the origins of ECMO patients required no pre-processing and included the aggregated numbers of pediatric and adult patients for individual administrative districts in Germany. In line with data protection regulations, cases numbering between 1 and 3 were subject to data redaction. For these instances, an assumed value of 2 was applied.

To delineate regions of potential undersupply or surplus provision, these data were transposed onto a color-coded Supply-Demand map. In the context of this study, ‘Supply’ refers to ECMO interventions as determined from hospital datasets. Conversely, ‘Demand’ characterizes the distribution of individual patients based on their place of residence. A positive difference shows regions where the number of hospital treatments exceeds the number of patients from the respective district.

### Statistical Analysis for the ECMO Patient Forecast

We provide a univariate one-period forecast for each of the 401 districts, foreign patients and patients with unknown origin, based on the observed time-series data. Our procedure was two-fold. To compare different models, we first conducted an Augmented Dickey Fuller (ADF) test to determine whether a time-series is stationary or not [26, 27]. For the stationary time-series, we employed the simple exponential smoothing method for forecasting [28, 29]. In the case of non-stationary time-series, we used the Holt linear trend method for forecasting [30]. Our second intended model was an Autoregressive Integrated Moving Average (ARIMA) for each time-series. To keep it feasible, we utilized a so-called Auto-ARIMA procedure [31], a Python package that provides the entire time-series analysis procedure in one line of code. With the Auto-ARIMA package, each time series resulted in its own autoregressive, moving average, and differencing order. A known limitation of both model classes is their construction for analyzing continuous data, not count data as in our work. Consequently, we rounded the estimated numbers. All statistical analyses were performed utilizing Python 3.9.14 packages Statsmodels [32], pmdarima [31], NumPy [33] and Pandas [34].

### Design of the Dashboard

The dashboard was created using Python and the open-source Plotly Dash application (Plotly Technologies Inc., Montreal, Quebec, Kanada) with contribution from CARTO© (CARTODB Inc., New York, NY, USA) and OpenStreetMap© (OpenStreetMap Foundation, Cambridge, UK). The use of Plotly Dash allowed a simple integration of interactive elements to enhance the user experience. The dashboard is publicly accessible online, and it was designed responsively to work cross-platform on a wide range of web-enabled devices.

## Results

**Fig. 1:**
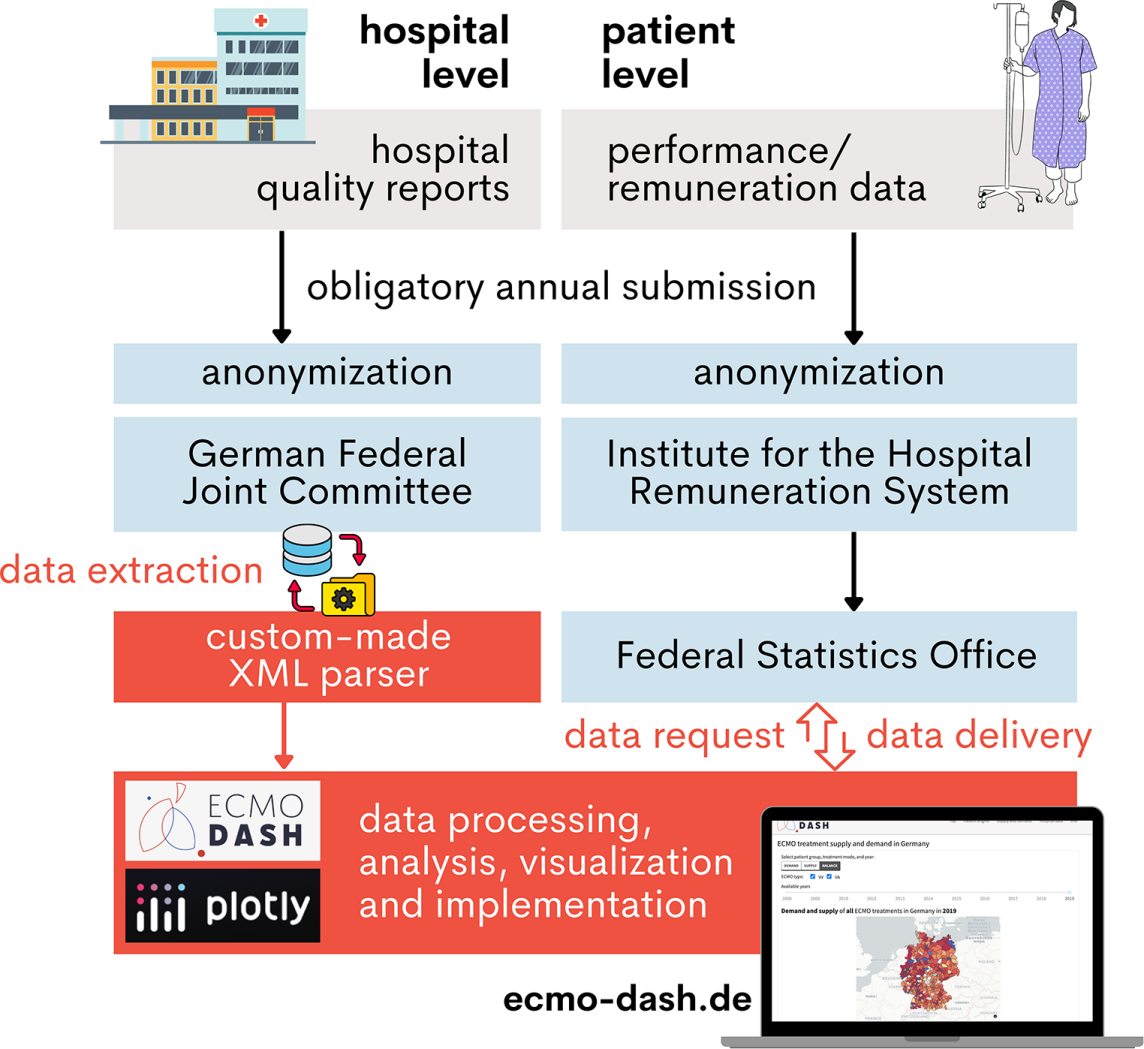
Data sources and processing pathway. Registered hospitals in Germany acquire hospital level and patient level data (grey) and are obliged to annually submit the data to official entities (blue). The data is extracted, processed, analyzed, and visualized (red) to implement it into the ECMO Dashboard

A total of 60,549 ECMO cases over 15 years (2005–2021) have been incorporated into the current iteration of the ECMO Dashboard (December 2023, Table 2). Anonymized data at both hospital and patient levels were extracted and/or requested from official entities in Germany. Subsequently, the fragmented data were technically merged, statistically analyzed, and graphically processed, resulting in a meaningful, aesthetically appealing, and user-intuitive tool for hospitals, policymakers, and the interested public (Fig. 1). The data are presented with unprecedented granularity for each of the 401 German city and county district (median area of 797.5 km^2^, ranging 35.7-5,495.6 km^2^; median population 170,632, ranging from 41,072 − 3,669,491 inhabitants in 2020) as well as for each of the hospitals performing ECMO (mean number of 213 per year, ranging from 48 in 2006 to 298 in 2020).

**Table 2:** Total number of ECMO patients and hospitals in different study periods (exemplary for two federal states)

Federal state		North-Rhine Westphalia (53 districts, 34,000 km^2^, 18 million inhabitants)			Berlin (1 district, 890 km^2^, 3.7 million inhabitants)		
		Year 2006	Year 2012	Year 2021	Year 2006	Year 2012	Year 2021
ECMO per 100,000 inhabitants	V-A	0	0.24	5.67	0	0,33	7.40
	V-V	0.88	3.48	8.32	0,12	2,28	8.1
Adult ECMO patients	V-A	0	38	988	0	10	260
	V-V	89	575	1461	4	62	296
Pediatric ECMO patients	V-A	0	4	29	0	1	12
	V-V	67	36	29	0	15	2
hospitals performing ECMO		12	53	91	1	7	13

**Fig. 2:**
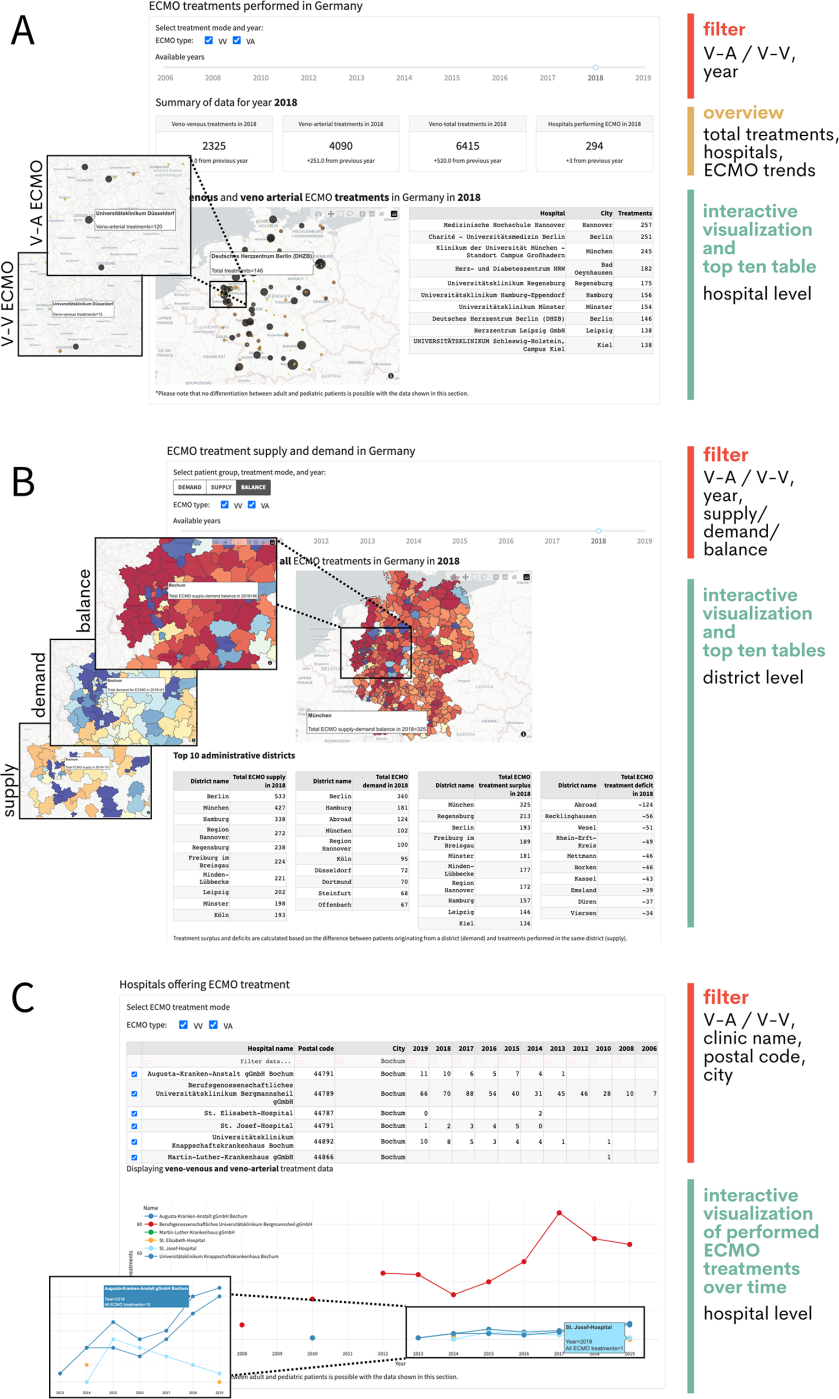
The ECMO Dashboard is an informative and interactive tool for broad use by professionals, policymakers, and the public. (**A**) Using convenient filter options (red), the ECMO supports performed in Germany are summarized (yellow) and presented (green). (**B**) The supply, demand, surplus, and deficit of ECMO support is explorable at district level (colors as in **B**). (**C**) ECMO volumes in German hospitals can be conveniently surveyed and displayed over time (colors as in **B** and **C**)

In more detail, the dashboard first presents the annual number of V-A and V-V ECMO support and the number of hospitals performing ECMO, including trends. An interactive map visualizes the numbers of ECMO support for each hospital and a nationwide top ten table is available for each year (Fig. 2). Further, three color-coded interactive district-level maps display (i) the origin of adult and pediatric V-A and V-V ECMO patients, (ii) the time series-based patient forecast for pediatric and adult V-V and V-A ECMO as well as (iii) the supply and demand of ECMO in each district (Fig. 2). The latter highlights potential areas of ECMO deficit (negative imbalance, red) or surplus (positive imbalance, blue). A positive imbalance indicates regions where the number of treatments is higher than the number of patients originating from the corresponding district. Accordingly, ECMO patients from other origin areas are transferred to the surplus district for medical, organizational, or other reasons. The four top-ten tables for this analysis present the districts with the nationwide highest ECMO supply, demand, surplus, and deficit, including patients from other countries treated in Germany (Abroad). The interactive maps feature tools for visual work. The box and lasso selection tools can be used to select hospitals or districts for highlighting them. The plots can be exported and downloaded as Portable Network Graphics and the view can easily be reset. Each map is supported by district-level top-ten tables (Fig. 2). For the origin of patients, it provides complementary information to the interactive map by listing the ten districts with the most ECMO patients per 10,000 inhabitants. Finally, it is possible to survey the development of ECMO numbers at hospital level and to compare the annual ECMO volume of different hospitals (Fig. 2).

The filter options of the ECMO Dashboard have been conveniently designed to offer an informative and interactive overview of ECMO therapy statistics, ranging from hospital-specific to citywide, district-wide, and nationwide levels. Notably, the filter sensitivity when searching for hospital names or cities can be adjusted so that the upper- and lower-case letters common in German are neglected.

## Discussion

This study uses a blend of national data to uniquely illustrate the landscape of ECMO support care across Germany, down to individual districts and hospitals via an accessible online dashboard. This aggregation and granular presentation of data facilitates the identification of regions with ECMO surpluses (districts that treat patients from other regions) and deficits (districts with no or insufficient ECMO capacity or medical expertise to treat severe ECMO cases). ECMO support in high-volume centers compared to low-volume centers was associated with better patient outcomes before and during the COVID-19 pandemic [15, 16, 35, 36]. However, a nuanced understanding of regional allocations and the overall prevalence of such a critical, personal-intensive procedure is vital to delineate the beneficial characteristics of high-volume ECMO centers and provide the safest intensive care to severely ill patients [36]. Building on the experience in England [37], where the CESAR trial [5] has highlighted the benefits of specialist ECMO centers for patient outcomes and healthcare costs, the ECMO Dashboard can be an important tool to enable the strategic alignment of ECMO care to proven models of centralized excellence. In the future, the ECMO Dashboard can be expanded to include additional important data for making scientifically informed and publicly auditable decisions about ECMO support efficiently and for the benefit of patients, medical staff, and society.

### Visualization of Healthcare Data

The ECMO Dashboard was designed and set up to provide information about ECMO support and patients nationwide in an interactive, comprehensive, and visually attractive way. We are not aware of comparable ECMO monitoring projects with high spatial granularity and easy access for all interested parties. Notably, the publication of hospital reimbursement data for non-commercial use is stipulated in § 21 of the German Hospital Remuneration Act (Krankenhausentgeldgesetz, KHEntgG) and likewise, these data serve as the basis for hospital planning by the responsible state authorities [38]. Germany in particular has a special position worldwide with the highest number of ECMO uses per inhabitant [39, 40]. The ECMO Dashboard presents publicly relevant but initially fragmented and difficult to use data in a way that makes it accessible to clinicians, scientists, healthcare management workers, policy makers, journalists, and the public. In this way, the ECMO Dashboard will increase the transparency of the German healthcare system, create incentives for its improvement, and promote trust among all stakeholders. Moreover, the easy availability and attractive presentation of health data is essential for convincing scientific communication about health phenomena and risks under normal circumstances and especially in times of crisis, such as pandemics [41]. The current version of the ECMO Dashboard is a tool for visualizing health data with a focus on the distribution and use of ECMO support in Germany. As ECMO support is not an index intervention, outcome analyses at hospital or patient level are not possible for data protection reasons.

### ECMO use in Germany during the COVID-19 Pandemic

The recent global health crisis underlines the importance of publishing health data as early as possible in a form that is understandable to all users. ECMO is a highly specialized procedure used to provide oxygenation and/or cardiac support in critically ill patients refractory to conventional treatment. The benefit of ECMO for critically ill COVID-19 patients remains controversial due to high in-hospital mortality rates of approximately 40–60% [35, 42–45]. In Germany, a large proportion of elderly COVID-19 patients (43% of patients were older than 60 years) received ECMO support in 2020–2021, with an unacceptably high mortality peaking at 84% for V-V ECMO in September 2021 [43]. Among other factors, age has been identified as a risk factor for ECMO patients and calls for stricter quality control and mechanisms to reduce futile invasive procedures in favor of patient-centered individual decisions have been made as early as 2021 [46]. Despite those numbers and expert statements, German decision makers did not limit the access to ECMO but continue offering a high, extrabudgetary reimbursement of this invasive technique, potentially creating financial incentives for its application [47]. Consistently, the ECMO Dashboard clearly depicts the steadily increasing numbers of ECMO support in Germany even before the pandemic (Table 2).

### ECMO Allocation and the ECMO Dashboard Prediction Tool

During the COVID-19 pandemic, substantial effort has been made in the United States to allocate mechanical ventilators in an ethically appropriate and medically most beneficial way [23, 24]. However, the bottleneck turned out to be ECMO. Patients were triaged, and the debate had to be expanded to this advanced intensive care technique with its unique ethical implications [20]. The shortage has been aggravated by the lack of a uniform system for allocating ECMO to hospitals [25]. Here parallels can be drawn to Germany, because an efficient and transparent management system of ECMO allocation does not exist. Thus, German patients and medical personnel will remain unprotected against the next pandemic without having implemented the lessons learned from COVID-19 worldwide. This is further exacerbated by the growing shortage of healthcare professionals, a trend that has intensified in the wake of the COVID-19 pandemic. The ECMO Dashboard provides a prediction tool for ECMO demand and supply. It will be continuously evaluated and improved to provide a basis for informed decisions regarding ECMO allocation under normal circumstances and in health crises. Nevertheless, this analytical tool, using current data, has inherent limitations due to the retrospective nature of the information, which is made available to researchers with a lag of up to two years. Although the COVID-19 pandemic spurred legislation mandating hospitals to submit billing data to the InEK more frequently [48], access to this data is currently restricted to the government and select agencies [49]. A policy shift to allow researchers earlier access to these data will improve analytical and prognostic tools for the benefit of patients and healthcare professionals, including refined forecasting of ECMO demand and supply for the ECMO Dashboard. Strikingly, it does not take a global pandemic of a new virus to threaten thousands of lives, as seasonal influenza has proven in many cases to be fatal for patients and challenging for healthcare workers. Therefore, policy makers and health management personnel are strongly encouraged to engage in proactive decision making and formulation of resource allocation strategies in collaboration with medical professionals, with a commitment to transparency to the public. The ECMO Dashboard provides an unparalleled foundation for these critical endeavors and can play a significant role in strengthening the nation’s critical care infrastructure.

### Limitations

These findings are based on secondary data initially generated for billing purposes [50]. Given the significant costs associated with ECMO procedures, accurate documentation is anticipated. However, inter-hospital patient transfers might lead to instances of double-counting. As ECMO support is no indexed medical intervention publicly available data and quality reports are subject to strict data protection laws.

Due to those data protection considerations, some data had to be censored, requiring us to work with average values. The datasets from DESTATIS and the quality reports from hospitals are published following a nearly two-year delay due to intricate administrative processes. Consequently, the projections are based on data from the past, which entails corresponding limitations.

## Conclusions

This study shows an innovative way of developing an informative and attractive healthcare management dashboard based on publicly available routine data. The ECMO Dashboard with its integrated prediction tool can be used by a variety of stakeholders to develop efficient and transparent healthcare management strategies, avoid ECMO shortage, and provide the best and safest intensive care under normal circumstances and during national health crises.

## Data Availability

The data used in this study is derived from two main sources. The first part is from the Federal Statistical Office of Germany, specifically the reimbursement data outlined under $21KHEntgG. While this data is freely available for scientific use, access is restricted and it is not publicly available to ensure confidentiality. The second part consists of data from the structured quality reports of hospitals (Qualitätsberichte der Krankenhäuser), collected by the German Federal Joint Committee (Gemeinsamer Bundesausschuss, G-BA). This data is publicly accessible and can be freely obtained. For access to the restricted data, researchers can submit a reasonable request, which, upon approval by the Federal Statistical Office of Germany, can be fulfilled by the authors.
